# Study on Machine Learning Models for Building Resilience Evaluation in Mountainous Area: A Case Study of Banan District, Chongqing, China

**DOI:** 10.3390/s22031163

**Published:** 2022-02-03

**Authors:** Chi Zhang, Haijia Wen, Mingyong Liao, Yu Lin, Yang Wu, Hui Zhang

**Affiliations:** 1 Key Laboratory of New Technology for Construction of Cities in Mountain Area, School of Civil Engineering, Ministry of Education, Chongqing 400044, China; zc158400@163.com (C.Z.); 201916131116@cqu.edu.cn (M.L.); FLyulin@163.com (Y.L.); 2 National Joint Engineering Research Center of Geohazards Prevention in the Reservoir Areas, Chongqing University, Chongqing 400045, China; 3 China Railway Guizhou Tourism and Culture Development Co., Ltd., Guiyang 550000, China; cquwy55555@163.com; 4 Investment Management Company of China Construction Fifth Bureau, Changsha 410007, China; 201816131097@cqu.edu.cn

**Keywords:** building resilience, machine learning, evaluation model, factor screening, model optimization

## Abstract

‘Resilience’ is a new concept in the research and application of urban construction. From the perspective of building adaptability in a mountainous environment and maintaining safety performance over time, this paper innovatively proposes machine learning methods for evaluating the resilience of buildings in a mountainous area. Firstly, after considering the comprehensive effects of geographical and geological conditions, meteorological and hydrological factors, environmental factors and building factors, the database of building resilience evaluation models in a mountainous area is constructed. Then, machine learning methods such as random forest and support vector machine are used to complete model training and optimization. Finally, the test data are substituted into models, and the models’ effects are verified by the confusion matrix. The results show the following: (1) Twelve dominant impact factors are screened. (2) Through the screening of dominant factors, the models are comprehensively optimized. (3) The accuracy of the optimization models based on random forest and support vector machine are both 97.4%, and the F1 scores are greater than 94.4%. Resilience has important implications for risk prevention and the control of buildings in a mountainous environment.

## 1. Introduction

‘Resilience’, derived from the Latin word ‘resilio’ [1], was first introduced into the field of ecology by Holling [2] in the 1970s. Subsequently, scholars have broadened the definition of ‘resilience’ to various research fields [3,4,5,6,7]. Different research and application fields have different definitions [8,9], corresponding to different evaluation methods. In the fields of engineering and construction, resilience is the ability to absorb or avoid damage without suffering complete failure and is an objective of design, maintenance and restoration for buildings and infrastructure, as well as communities [10,11]. At present, there are different research methods regarding resilient cities and resilient communities [12], but most of them consider the assets (economy, society, environment and infrastructure) and functions (social capital, community function, transportation and communication links and planning) of the community. Buildings, an important part of infrastructure, are inevitably damaged to varying degrees in the actual use and operation process, resulting in property and even life loss. The building resilience in a mountainous area [13] can be understood as the ability of buildings that are under the conditions of the mountainous environment to still maintain their normal function and resist damage or to recover from the comprehensive effects of its various attributes, natural environment and the passage of time.

Many scholars have carried out a series of studies from different perspectives on the issue of building resilience. A resilience-based performance evaluation [14] is employed within a multiobjective optimization methodology for the design optimization of 4, 7, 10 and 15-story buildings under seismic hazard using both life span and conditional analyses. Himoto et al. [15] developed a computational framework using a multi-layer zone model to evaluate the fire resilience of buildings. Dong et al. [16] proposed a method to evaluate the seismic resilience of a steel structure considering economic, social and environmental aspects. For key infrastructure such as hospitals, Bruneau [17] explored the operational and physical resilience of acute care facilities.

In recent years, machine learning algorithms have attracted increasing attention in the field of risk assessment management [18,19,20,21]. Riedel et al. [22] carried out seismic vulnerability assessment of urban environments in moderate-to-low seismic hazard regions using association rule learning and support vector machine methods. Xie et al. [23] reviewed the promise of implementing machine learning in earthquake engineering. Some scholars have also used machine learning methods to study the building classification problem [24,25]. Several works [26] have described a hybrid information fusion approach to quantitatively evaluate the seismic resilience of Nepal by formulating nine indicators at the geological, building and social dimensions. Mangalathu et al. [27] used discriminant analysis, k-nearest neighbors, decision trees and random forests to study the damage degree of houses after an earthquake. Zhang et al. [28] used the support vector machine to study the physical resilience evaluation of landslide disasters in cities.

At present, the research on building resilience mainly considers the single factor effect represented by earthquakes. Moreover, it mainly considers the building structure, ignoring the complexity of the interaction between time and the internal and external factors of buildings combined. Studies on resilience in a mountainous environment are limited. Barua et al. [29] studied the resilience of rural mountain communities in relation to climate change and poverty in a mountainous region of India. Mountains are among the regions most affected by climate change [30,31], and climatic factors have an impact on building resilience. Meanwhile, the geographical and geological conditions in a mountainous environment are complex, and natural disasters such as collapse and landslide are likely to occur. Therefore, it is necessary to study the building resilience in mountainous environments specifically. With reference to the provisions of the technical guidelines for rural housing safety appraisal [32] and standards for dangerous building appraisal [33] in China, this paper classifies buildings as Grade I, II and III with regards to building resilience in mountainous areas (Table 1).

With the development of spatial and information technologies, a large amount of temporal and spatial data can be collected, processed and presented [34]. The objective of this study is to develop models for evaluating the resilience of mountainous buildings that take into account the combined effects of the various internal building properties, the natural environment and the passage of time. Firstly, the evaluation index system of building resilience in a mountainous area is constructed, and the dominant factors are screened using the feature recursive elimination method. Secondly, the building resilience models are completed by machine learning methods, including random forest and support vector machine, and the model evaluations are performed by confusion matrix. Finally, the predicted data are substituted into the model to obtain the classification evaluation of building resilience in the area to be studied. The original determination of the resilience grade requires a personal visit by professionals, which is labor-intensive. Through the machine learning method, the building resilience rating of the area to be studied can be determined quickly without visiting the site and without spending considerable time and manpower. This method provides additional value and reference significance in risk prevention and the control of buildings in a mountainous environment.

## 2. Study Area

Banan District is located in the south of Chongqing central city, with an area of 1825 square kilometers and a built-up area of 84.5 square kilometers. It is a typical mountainous county. The gap between urban and rural areas is large, and there are huge differences in the quality of buildings and their ability to withstand natural environmental disasters. The selection of Banan District as the research area of building resilience in a mountainous area has high theoretical value and practical significance.

**Figure 1:**
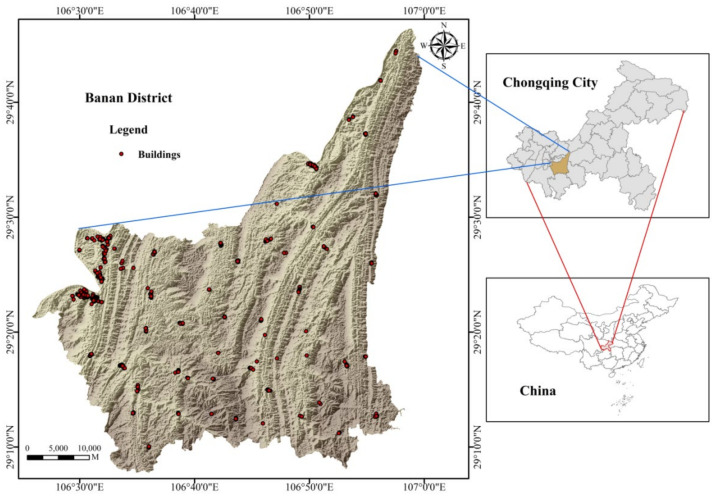
Location and buildings’ distribution of Banan District.

Our research team and Chongqing Municipal Public Housing Administration Office collected and analyzed data through field research. They obtained data from 1387 buildings in Banan District, including 122 buildings with resilience grade Ⅰ, 352 buildings with resilience grade Ⅱ and 913 buildings with resilience grade Ⅲ. Figure 1 shows the geographical location of Banan District and the distribution of surveyed buildings.

## 3. Data and Methods

### 3.1. Data

#### 3.1.1. Data Selection

The resilience of buildings in a mountainous area is affected by a combination of various internal and external factors, such as geographical and geological factors, meteorological and hydrological factors, environmental factors and building factors [35,36]. Based on the above four dimensions, 21 factors were select to establish the factor database of building resilience evaluation in a mountainous area. They are as follows: elevation, slope, slope aspect, slope position, curvature, plan curvature, profile curvature, micro-landform [37], terrain humidity index (TWI), terrain roughness index (TRI), lithology, average annual rainfall (AAR), aridity, temperature, distance from fault, distance from roads, distance from rivers, building structure, construction time, building storey and building category.

Geographical and geological factors fully consider the particularity of mountain building topography. Elevation affects climate and human activities. Slope affects the stress distribution of rocks and soil. Slope aspect and slope position influence hydrogeology. Curvature affects soil erosion through water flow on the slope. Plan curvature refers to the change rate of surface aspect at any point on the ground. Section curvature refers to the change rate of surface slope at any point on the ground. Micro-landform is a small terrain fluctuation with the surface complexity of large geomorphology, which affects the strength and weathering degree of rock and soil. TWI considers comprehensively the influence of terrain and soil characteristics on water distribution. TRI refers to the degree of concavity of the soil surface, reflecting the effects of wind and water erosion on the soil. Due to the different formation times and weathering degrees, the bearing capacity of different lithologies is also different. Meteorological and hydrological factors take into account the effect of time, average annual rainfall, aridity and temperature. They affect the durability of buildings. Environmental factors affect the original rock stress and slope stability of buildings through natural (fault, rivers) and human engineering activities (roads). Building factors are internal factors that lead to differences in housing quality and ability to resist natural disasters. Different building structures, categories, storeys and construction times lead to different building materials, weights and aging degrees.

#### 3.1.2. Data Source

Data were obtained from 1387 buildings in Banan District, including the building structure, construction time, building storey and building category, through field investigation by the School of Civil Engineering of Chongqing University and Chongqing Municipal Public Housing Administration Office. DEM of ArcGIS was used to extract and process the data of slope, slope aspect, slope position, curvature, plan curvature, profile curvature, micro-landform, TWI and TRI. Other data sources, types and scale are shown in Table 2.

#### 3.1.3. Data Processing

The factors were quantified and reclassified. The continuous factors such as elevation, slope, curvature, plan curvature, profile curvature, TWI, TRI, average annual rainfall, aridity and temperature were classified by ArcGIS natural breaks method (Jenks). The 360° was divided into eight regions on average, and the flat was assigned separately, so the slope aspect was divided into nine categories. The distances from fault, roads and rivers were obtained by multiple ring buffer of fault, roads and rivers, respectively, through ArcGIS. The qualitative factors such as slope position, micro-landform, lithology, building structure and building category were classified according to their respective characteristics. In this paper, building structure categories were distinguished mainly based on building materials. The structures, which include timber structure, adobe–timber structure, brick–timber structure, brick–concrete structure, as well as steel and reinforced concrete structure, were named directly using the names of materials. The simple structure referred to the building with simple materials such as brick or wood panels. In addition, only a few buildings built of stone–timber and stone–concrete materials were situated in the study area, which were collectively referred to as mixed structures. The construction time was grouped by a minimum of ten years based on data distribution. The building storey adopted the original data. According to their different uses, the buildings in this paper were divided into several categories, including residential building, commercial building, teaching building, auxiliary building and other building. Auxiliary buildings refer to buildings with auxiliary functions as their main purpose. For rural areas, they include buildings such as toilets and those used for storage of agricultural production tools, breeding of farm animals, drying and storage of food crops, etc. For urban areas, they comprise buildings such as public toilets, gatehouses, those used for auxiliary housing and public services, etc. Buildings that did not meet the above criteria were classified as other buildings. The reclassification of impact factors is shown in Table 3.

After reclassification, the impact factors’ data were normalized. All values were normalized to the distribution between (0,1). All factors were in the same order of magnitude in order to facilitate correct and rapid modelling. The normalization formula is denoted as follows

(1) $$X^{*}=\left(X-X_{min}\right)/\left(X_{max}-X_{min}\right)$$

In the formula, $X^{*}$ is the normalized data, X is the original data, $X_{max}$ and $X_{min}$ are the maximum and minimum of the data, respectively.

**Figure 2:**
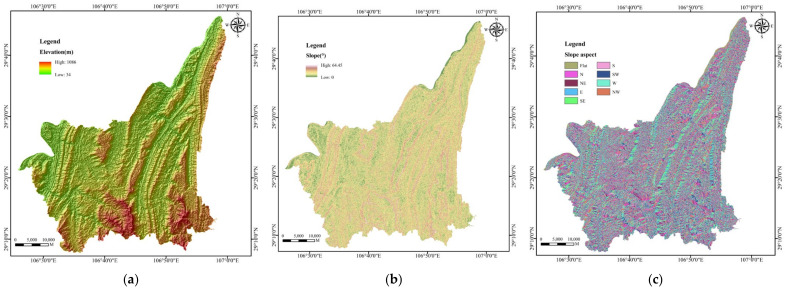

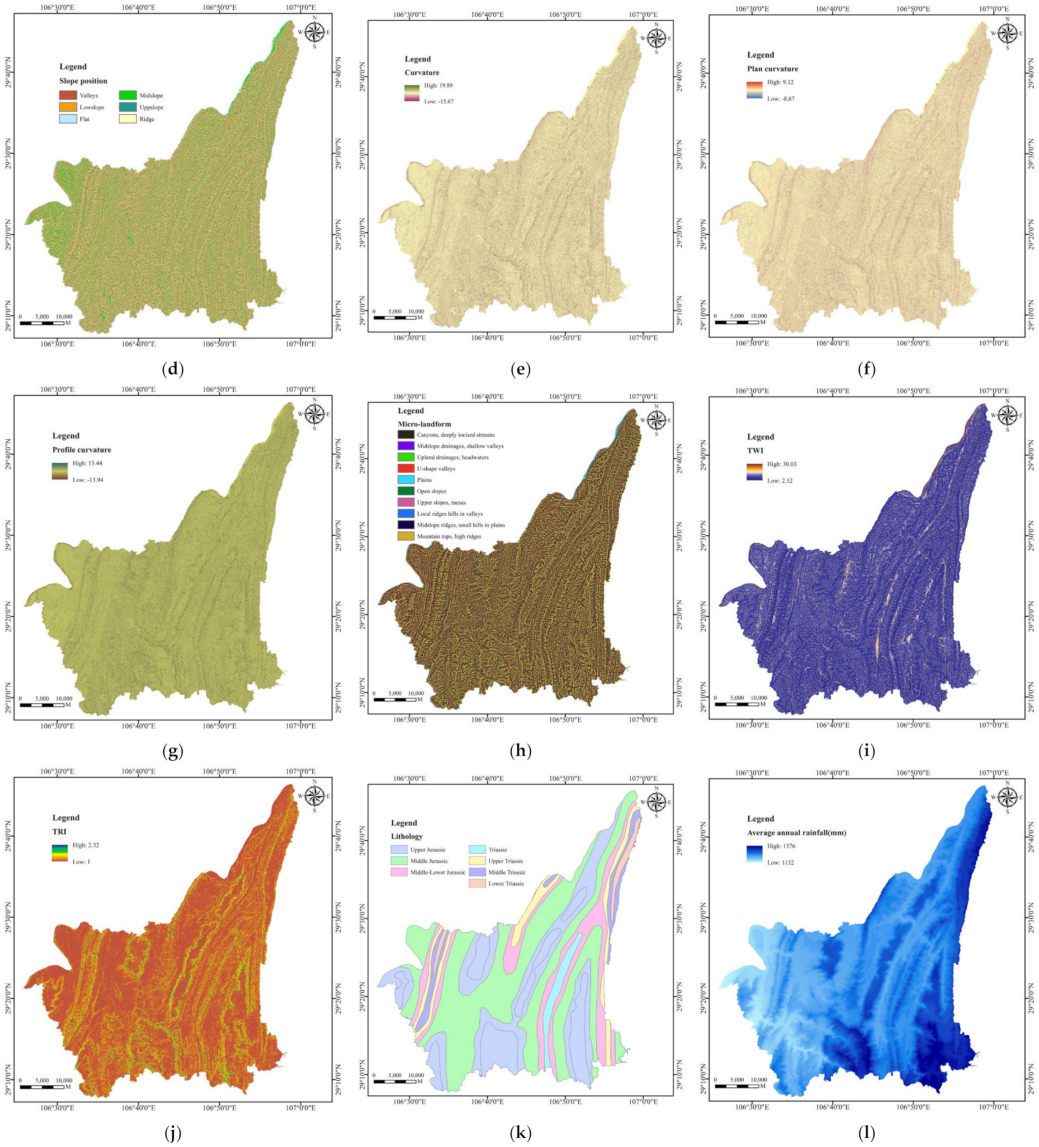

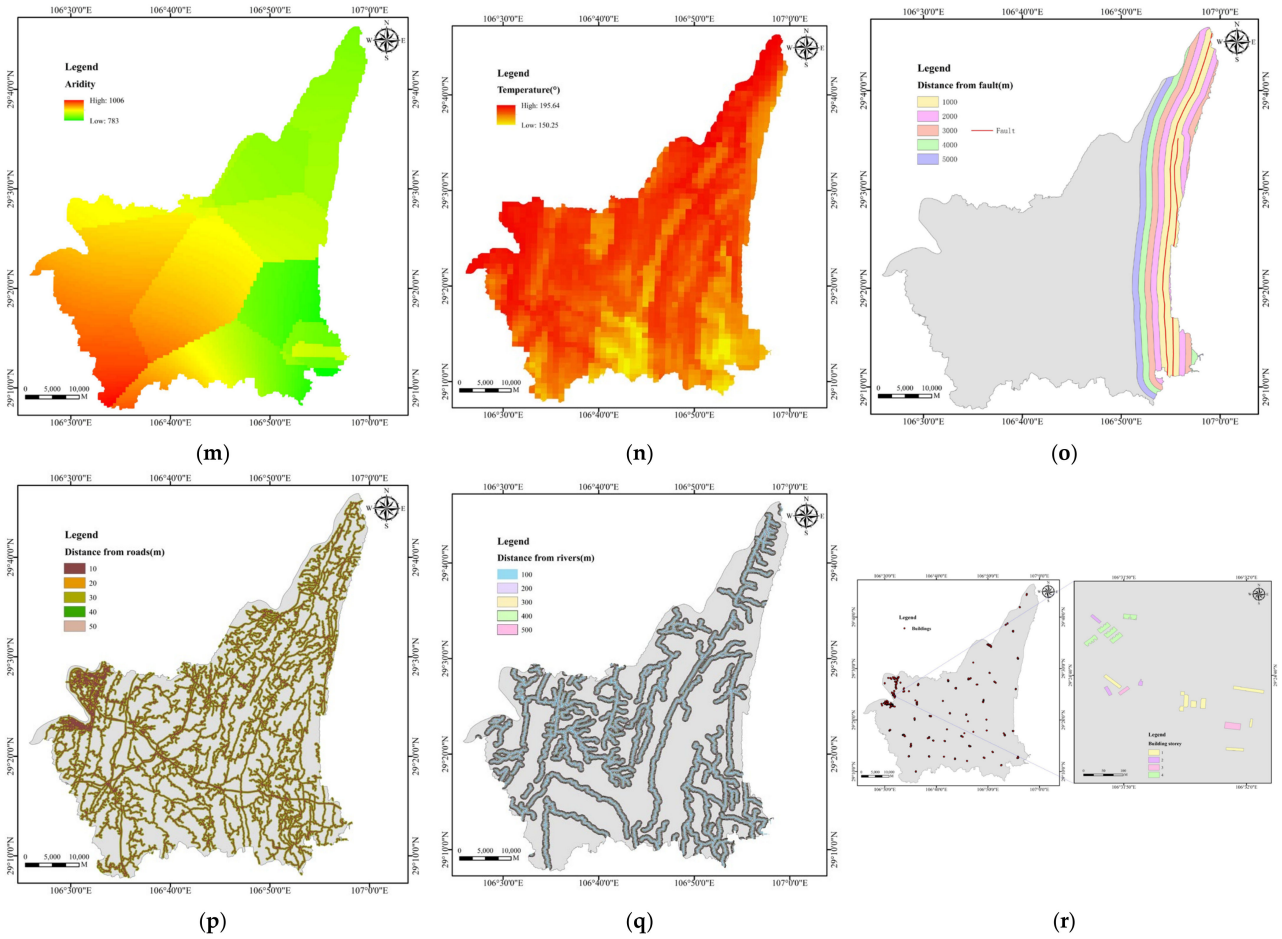
Thematic layers of impact factors: (**a**) Elevation; (**b**) Slope; (**c**) Slope aspect; (**d**) Slope position; (**e**) Curvature; (**f**) Plan curvature; (**g**) Profile curvature; (**h**) Micro-landform; (**i**) TWI; (**j**) TRI; (**k**) Lithology; (**l**) Average annual rainfall; (**m**) Aridity; (**n**) Temperature; (**o**) Distance from fault; (**p**) Distance from roads; (**q**) Distance from rivers; (**r**) Building factors.

For better data management and visual representation, the corresponding thematic layers were constructed by ArcGIS, as shown in Figure 2. The specific distribution of the impact factors of geographical and geological factors, meteorological and hydrological factors, environmental factors and building factors can be displayed visually. However, due to the small building area and the large study area, the buildings were only shown as points under the full view of the study area. Figure 2r shows the construction of building factor layers of ArcGIS with the building storey as an example. The attribute table corresponding to the building recorded all the information of each building, including building structure, construction time, building storey and building category. Changing its fields in properties switches it to other building factor layers.

### 3.2. Methodology

#### 3.2.1. Random Forest

Random forest (RF) is a data mining algorithm that contains multiple decision trees. Based on each decision tree, the final classification result is obtained by voting [38]. Random forest model has strong robustness and accuracy in data processing. This study selected random forest as one of the processing algorithms of the model.

By calling the random forest program package through R language, the data obtained from the 1387 buildings containing all the information of influencing factors in the study area were regarded as the total samples, which were randomly divided into 971 training samples and 416 test samples according to the ratio of 7:3. The ratio of 7:3 is an empirical value that has been used by many researchers. The optimal parameter *mtry* was selected by cyclic iteration, and it was substituted into the code to view the error stability of the model and find the optimal *ntree*. *Mtry* refers to the number of variables used for binary trees in nodes, and *ntree* refers to the number of decision trees contained in random forests.

#### 3.2.2. Support Vector Machine

In recent years, many scholars have carried out in-depth research on disaster risk assessment using the support vector machine (SVM) algorithm [39,40,41]. The basic idea is to use kernel function to project nonlinear separable samples into high-dimensional space to construct linear separable samples. According to the spatial distribution of sample features, the optimal hyperplane solution with the farthest distance between the two groups of classifications was found, so as to correctly divide the data set. This project used the ksvm function of kernlab software package [42]. For the three-classification problem, ksvm used ‘one-to-one’ method to construct three secondary classifiers by permutation and combination, and judged the resilience grade of buildings in mountainous area by voting. In this study, the SVM model was selected as another prediction model to measure the reliability of the RF model.

In the support vector machine model, the parameters were also optimized first. The kernlab package was called by R language, and the optimal parameter combination *sigma* and *C* value were selected in the for-loop iteration through the tenfold cross validation. *Sigma* determines the width of the kernel function, and *C* refers to the tolerance of allowing classification errors. Then, the above optimal parameter combination was substituted to establish the model.

#### 3.2.3. Feature Recursive Elimination

In machine learning, not all the results of variable prediction are related. Some irrelevant variables may have a negative impact on the model prediction accuracy. Through feature selection, the results of model effect optimization can be achieved. The main idea of feature recursive elimination method is to eliminate the factor with the smallest ranking criterion score at each time on the basis of all the initial influencing factors and to construct the model repeatedly until the final feature set is obtained [43]; the ranking of features is obtained at the same time.

#### 3.2.4. Model Evaluation Methods

In this paper, the resilience of buildings in mountainous area is divided into grades I, II and III. The prediction effect is analyzed by confusion matrix analysis model. The confusion matrix is an error matrix that measures the predicted and actual values, which can be used to evaluate the accuracy and stability of machine learning algorithms. In order to simplify the expression, the data are referred to by the combination of the real value before and the predicted value after (Table 4). *N_ij_* (*I* = 1,2,3; *j* = 1,2, 3) represents the number of samples that actually belong to *i* but are predicted to be *j* [44].

Accuracy rate refers to the proportion of samples with correct prediction, considering the total samples. It is the most basic, intuitive and simple method to measure the evaluation effect of classification model. Precision refers to the proportion of the true values of a grade, considering all the samples predicted as a certain grade, reflecting the precision of the model prediction. Recall rate represents the proportion that is predicted accurately in the actual sample of a certain grade. In order to take both precision and recall into account, the harmonic mean F1 score was used as another reference index. The calculation formulas are as follows

(2) $$Accuracy=\overset{3}{\underset{i=1}{\sum }}N_{ii}/\overset{3}{\underset{i=1}{\sum }}\overset{3}{\underset{j=1}{\sum }}N_{ij}$$

(3) $$Precision_{i}=N_{ii}/\overset{3}{\underset{k=1}{\sum }}N_{ki}$$

(4) $$Recall_{i}=N_{ii}/\overset{3}{\underset{k=1}{\sum }}N_{ik}$$

(5) $$F_{1}score=2\times Precision_{i}\times Recall_{i}/\left(Precision_{i}+Recall_{i}\right)$$

## 4. Results and Discussion

### 4.1. Optimization Models of Building Resilience Based on Dominant Factors

#### 4.1.1. Screening of Dominant Factors

**Figure 3:**
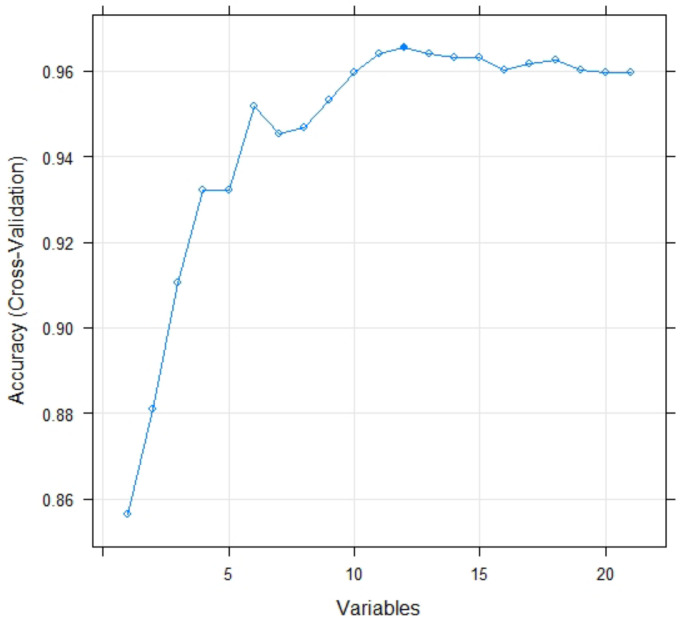
Screening diagram of dominant factors by using FRE.

This paper selected the feature recursive elimination (FRE) method to filter the dominant factors for model optimization. Based on the R language call code, when the number of impact factors was 12, the model worked best (Figure 3). The dominant factors screened were elevation, lithology, TRI, aridity, temperature, average annual rainfall, distance from roads, distance from rivers, building structure, building category, construction time and building storey.

#### 4.1.2. Optimization models’ results of building resilience based on dominant factors

**Figure 4:**
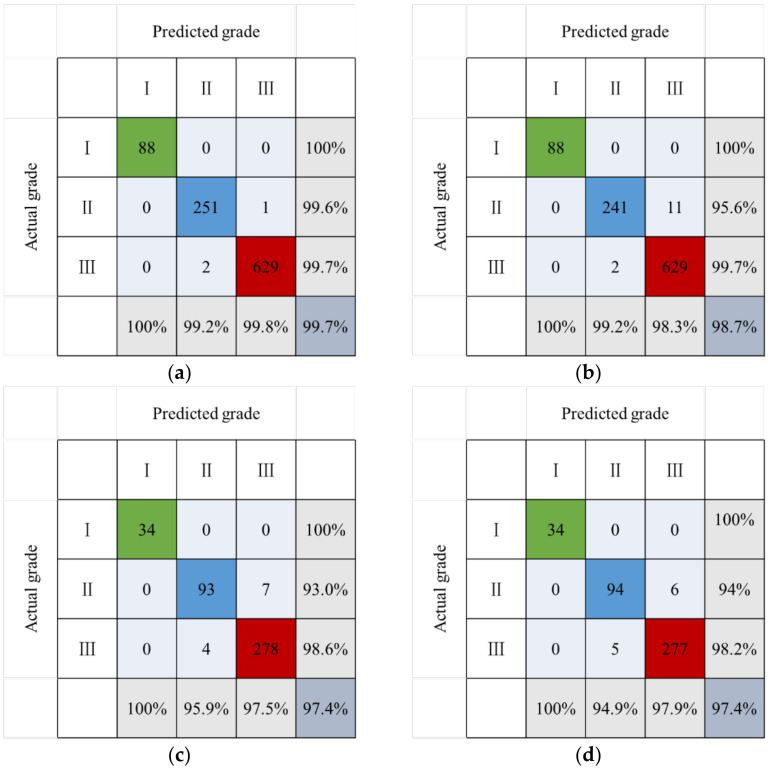

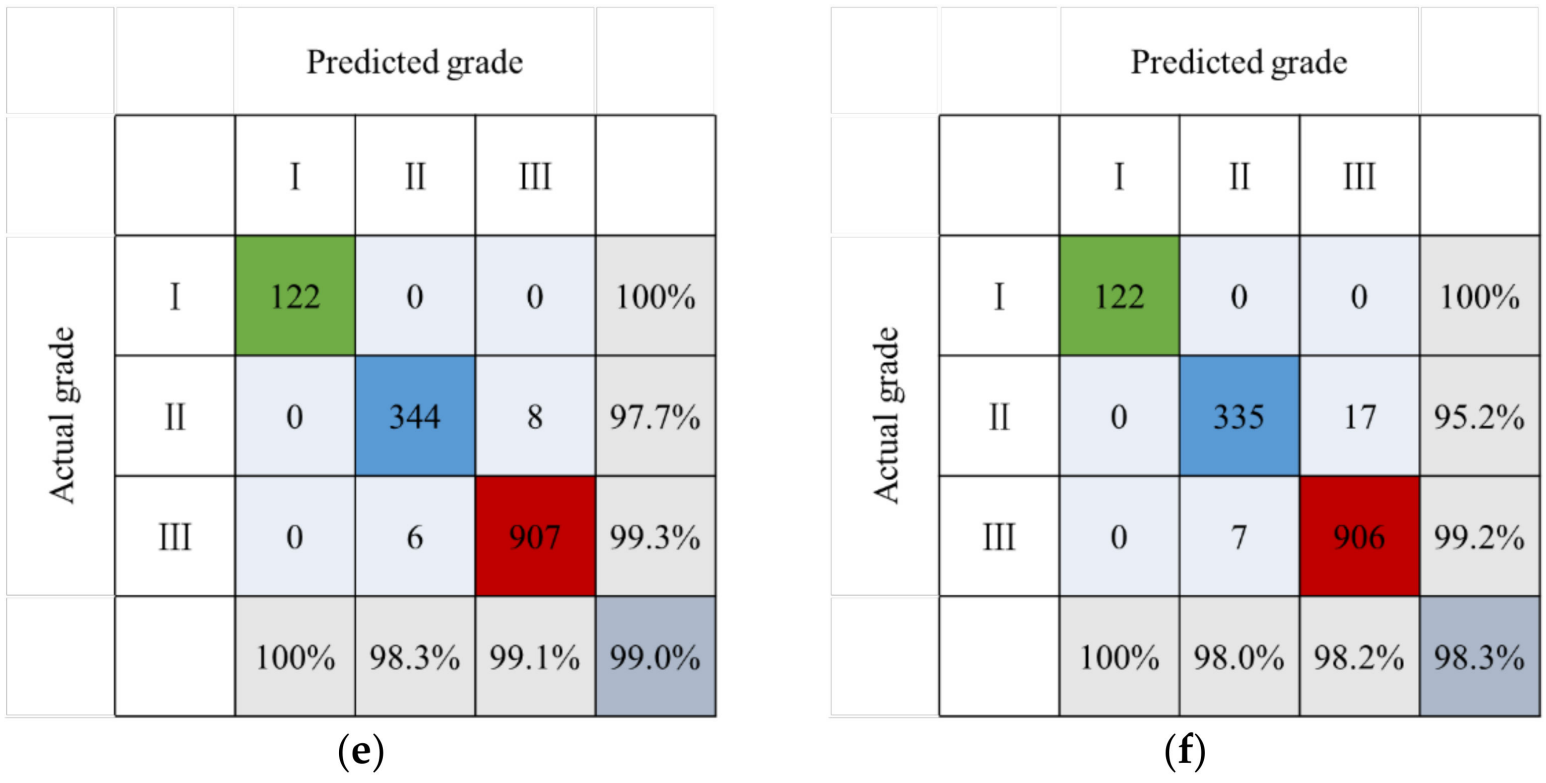
Confusion matrices of optimization models based on machine learning: (**a**) Training samples-RF; (**b**) Training samples-SVM; (**c**) Test samples-RF; (**d**) Test samples-SVM; (**e**) Total samples-RF; (**f**) Total samples-SVM.

The dominant factor was used as input layer, while mountain building resilience grade was used as output layer. After debugging, in the random forest model, the optimal parameters *mtry* = 8 and *ntree* = 1000 were selected. In the support vector machine model, the optimal parameter combination kpar = list (*sigma* = 0.21) and *C* = 5 were selected. Thus, the confusion matrix of the prediction results of the training samples, test samples and total samples based on the random forest and support vector machine algorithm was obtained (Figure 4). The nine data in the matrix center are the direct output results of the confusion matrix. The three data on the left side of the last line are the precision of building resilience grades I, II and III. The three data above the last column are the recall of their respective grades. The data in the bottom-right corner are the model accuracy.

Based on random forest and support vector machine, the accuracies of the building resilience optimization models in mountainous area are calculated using training samples, test samples and total samples, respectively. For training samples, the model accuracies based on random forest and support vector machine are 99.7% and 98.7%, respectively. For test samples, both are 97.4%; for the total samples, they are 99.0% and 98.3%, respectively. Accuracy is a metric in confusion matrix for evaluating the mountainous building resilience model, and a larger accuracy rate indicates a better model. Observing the precision of the model in the test samples, RF and SVM are very good in the prediction of grade I buildings. In the prediction of grade II buildings, the random forest model is better than the support vector machine model. The support vector machine model is better in the prediction of grade III buildings. All precisions are above 94.9%. Observing the recall of the model in the test samples, RF and SVM are very good in the prediction of grade I buildings. In the prediction of grade II buildings, the SVM model is better than the RF model, while the RF model is better in the prediction of grade III buildings. All recalls are above 93.0%. The F1 score comprehensively considers the precision and recall. The two models have good prediction effect on grade I buildings. There are occasional misjudgments in grade II and grade III buildings, but all values are greater than 94.4%.

In summary, the prediction accuracy, precision, recall and F1 scores of random forest and support vector machine are high, which proves that the machine learning method is reliable for resilience evaluation of buildings in mountainous area.

### 4.2. Optimization Effect Comparison

The training samples were used to construct the model, and all the evaluation indexes in the confusion matrix are the maximum values. The total samples cover part of the modelling data, and the values are between the training samples and the test samples. The test samples do not participate in model building, but can better detect model performance. The effects of model optimization are analyzed for the test samples.

**Figure 5:**
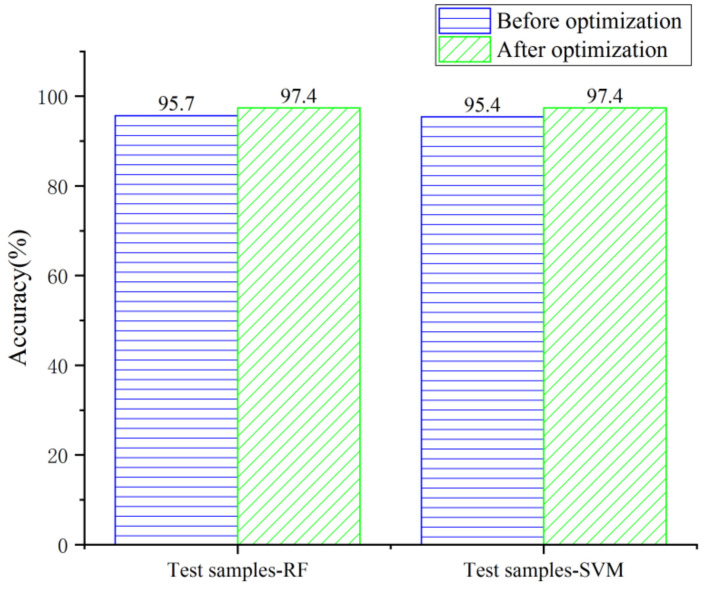
Comparison of test samples’ accuracy before and after optimization.

Accuracy is the most basic evaluation index of the model. After optimization, the accuracy of the random forest model was improved from 95.7% to 97.4%, and the accuracy of the support vector machine model was improved from 95.4% to 97.4% (Figure 5).

**Figure 6:**
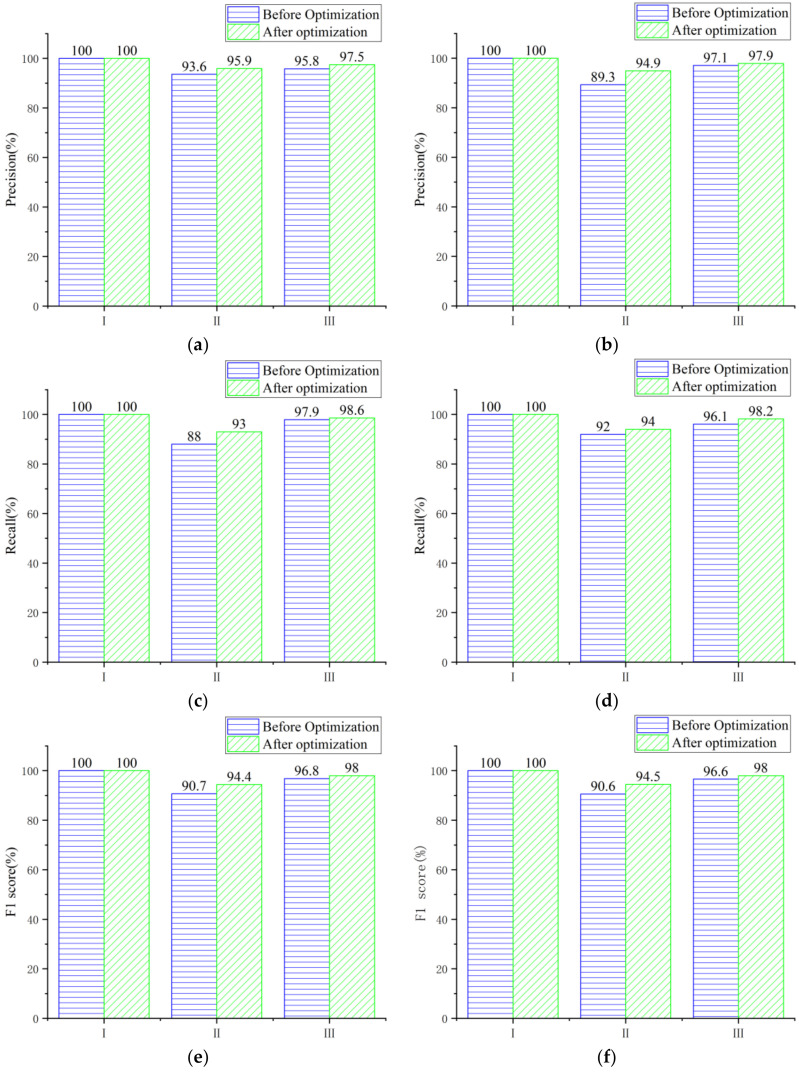
Comparison of test samples’ evaluation indexes before and after optimization: (**a**) Precision-RF; (**b**) Precision-SVM; (**c**) Recall-RF; (**d**) Recall–SVM; (**e**) F_1_ score-RF; (**f**) F_1_ score-SVM.

As shown in Figure 6, compared with the pre-optimization state, the minimum value of each index of the model based on the dominant factors’ screening improved from 88% to 93%. The model effect was comprehensively improved. The best optimization effect of SVM was that the precision of grade II increased by 5.6%, and the best optimization effect of RF was that the recall of grade II increased by 5%. The range of variation of indicators for each building’s resilience grade was inconsistent, which may be due to the quantity and quality of the data themselves. The two machine learning algorithms have different emphases on model optimization but the effects are remarkable.

### 4.3. Discussion

#### 4.3.1. Comparison of Two Machine Learning Models

In the test samples, the evaluation indexes of RF and SVM optimization models were compared (Table 5). It was observed that the two machine learning methods have the same evaluation results for accuracy rate, recall, F1 score of grade I buildings and F1 score of grade III buildings. The RF model is superior to the SVM model in the evaluation of the precision of grade II buildings and the recall of grade III buildings. The SVM model is better than the RF model in the evaluation of grade III buildings’ precision, grade II buildings’ recall and F1 score. Both methods have advantages and disadvantages in each evaluation index, but the absolute value of the difference does not exceed 1%. It was proved that RF and SVM are reliable in the evaluation of building resilience in a mountainous area.

#### 4.3.2. Importance of Resilience Impact Factors

The importance ranking of impact factors reflects the contribution of variables to the resilience evaluation model of buildings in a mountainous area. Random forest provides two methods for ranking the importance of features: Mean Decrease Accuracy (MDA) and Mean Decrease Gini (MDG) [45]. MDA is the change in the error rate of model results caused by disrupting the value of an impact factor in the test set. MDG is the sum of all decreases in Gini impurity due to a given variable. Based on the study by Han et al. [46], this paper combined MDA and MDG for a comprehensive measure. The 12 variables were assigned scores of 12, 11,…, 2, and 1 based on the values of MDA and MDG from highest to lowest, respectively. The scores obtained from both were then added and re-ranked to obtain the combined ranking results of the importance of the influencing factors (Table 6).

**Figure 7:**
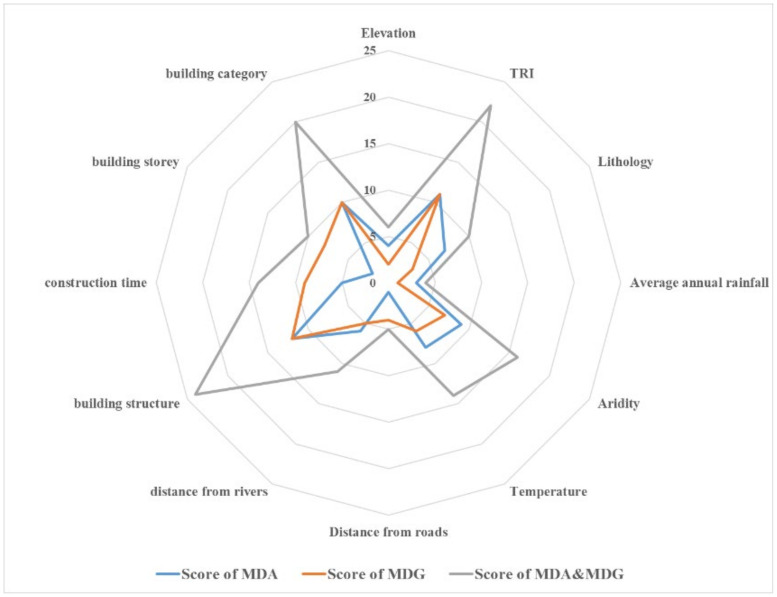
Impact factors’ assignment score chart.

The results are in the following order: building structure, TRI, building category, aridity, construction time, temperature, distance from rivers, lithology, building storey, elevation, distance from roads and average annual rainfall. For the optimized dominant factor index, all building factors, all meteorological and hydrological factors, three geographical and geological factors, and two environmental factors are selected, which are comprehensive and representative. The alternate arrangement of internal and external factors fully illustrates the necessity of exploring the combined effect of various factors on buildings in a mountainous area. Figure 7 shows the degree of importance of each impact factor clearly.

#### 4.3.3. Model Improvement Options

The building resilience models in a mountainous area work well, but there is still room for improvement. Regarding improvement from the perspective of impact factors, more impact factors such as extreme temperature should be considered in the preliminary selection stage. Moreover, regarding improvement from the perspective of machine learning methods, data imbalance should be the focus in subsequent research. The classification algorithm will produce a certain bias when processing the data set according to the amount of data in different categories. For unbalanced data sets, assigning different weights for processing should be considered.

## 5. Conclusions

Based on machine learning, this paper proposed a resilience evaluation method for buildings in a mountainous area. Considering the multi-dimensional effects of geographical and geological conditions, meteorological and hydrological factors, environmental factors and building factors, the database of impact factors was constructed. The models were trained and optimized by machine learning methods, including random forest and support vector machine, and the resilience evaluation models of buildings in a mountainous area were established. Then, the predicted data were substituted into the model to obtain the classification evaluation of building resilience in the area to be studied.

(1) By combining MDA and MDG to form a comprehensive measure, the impact factors of the optimization models were ranked in order of importance: building structure, TRI, building category, aridity, construction time, temperature, distance from rivers, lithology, building storey, elevation, distance from roads and average annual rainfall. In the respective rankings of MDA and MDG, the impact factors in the top three rankings are the same, and the remaining impact factors tend to differ between the two. The alternate arrangement of internal and external factors fully illustrates the necessity of exploring the combined effect of various factors on buildings in a mountainous area.
(2) Through the screening of dominant factors, the minimum value of each index in the model test sets was increased from 88% to 93%, the models were comprehensively optimized, demonstrating the need for factor screening. The two machine learning algorithms have different emphases on model optimization, but the effects were remarkable.
(3) The accuracy of the optimization models based on random forest and support vector machine were both 97.4%, and the F1 scores were greater than 94.4%, which proves that the machine learning method is reliable for resilience evaluation of buildings in a mountainous area. This study has the advantages of accuracy, efficiency and visualization. It provides additional value and reference significance in risk prevention and the control of mountainous environment building construction.

## Figures and Tables

**Table 1:** Resilience grades of typical buildings.

Building Resilience Grades	Grade I	Grade II	Grade III
Grading criteria	Buildings whose structure is basically safe for use	Local dangerous buildings in which a part of the load-bearing structure cannot meet the requirements of safe use.	Whole dangerous buildings in which the load-bearing structure cannot meet the requirements of safe use.
Pictures from the scene	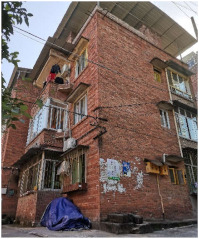	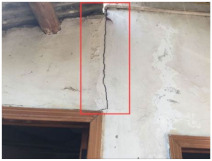	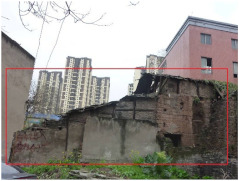

**Table 2:** Statistics of data sources.

Category	Data	Data Source	Scale
Geographical and geological factors	Elevation	ASTER	30 m
	Lithology	National Geological Archives of China	1:200000
Meteorological and hydrological factors	Average annual rainfall	China Meteorological Data Service Centre-Resource and Environment Science and Data Center	30 m
	Aridity	Resource and Environment Science and Data Center	500 m
	Temperature	Resource and Environment Science and Data Center	1000 m
Environmental factors	Fault	National Geological Archives of China	1:200,000
	Roads	Google remote sensing images	1:250,000
	Rivers	Google remote sensing images	1:250,000

**Table 3:** Reclassification of impact Factors.

Category	Impact Factors	Number of Categories	Classification Criteria
Geographical and geological factors	Elevation (m)	9	(1) ≤244; (2) 244~312; (3) 312~377; (4) 377~448; (5) 448~525; (6) 525~605; (7) 605~691; (8) 691~802; (9) ≥802
	Slope (°)	9	(1) ≤5.03°; (2) 5.03°~8.70°; (3) 8.70°~12.33°; (4) 12.33°~16.07°; (5) 16.07°~20.08°; (6) 20.08~24.57; (7) 24.57~29.88; (8) 2 9.88~36.94; (9) ≥36.94
	Slope aspect	9	(1) Flat; (2) N; (3) NE; (4) E; (5) SE; (6) S; (7) SW; (8) W; (9) NW
	Slope position	6	(1) Valleys; (2) Lowslope; (3) Flat; (4) Midslope; (5) Uppslope; (6) Ridge
	Curvature	9	(1) ≤−4.09; (2) −4.09~−2.46; (3) −2.46~−1.29; (4) −1.29~−0.47; (5) −0.47~0.35; (6) 0.35~1.17; (7) 1.17~2.24; (8) 2.24~4.09; (9) ≥4.09
	Plan curvature	9	(1) ≤−1.97; (2) −1.97~−1.21; (3) −1.21~−0.65; (4) −0.65~−0.23; (5) −0.23~0.19; (6) 0.19~0.61; (7) 0.61~1.17; (8) 1.17~2.00; (9) ≥2.00
	Profile curvature	9	(1) ≤−2.88; (2) −2.88~−1.70; (3) −1.70~−0.95; (4) −0.95–0.41; (5) −0.41~0.12; (6) 0.12~0.66; (7) 0.66~1.41; (8) 1.41~2.59; (9) ≥2.59
	Micro-landform	10	(1) Canyons, deeply incised streams; (2) Midslope drainages, shallow valleys; (3) Upland drainages, headwaters; (4) U-shape valleys; (5) Plains; (6) Open slopes; (7) Upper slopes, mesas; (8) Local ridges hills in valleys; (9) Midslope ridges, small hills in plains; (10) Mountain tops, high ridges
	TWI	9	(1) ≤4.68; (2) 4.68~5.87; (3) 5.87~7.16; (4) 7.16~8.56; (5) 8.56~10.18; (6) 10.18~12.12; (7) 12.12~14.71; (8) 14.71~17.95; (9) ≥17.95
	TRI	9	(1) ≤1.018; (2) 1.018~1.041; (3) 1.041~1.071; (4) 1.071~1.108; (5) 1.108~1.155; (6) 1.155~1.217; (7) 1.217~1.304; (8) 1.304~1.450; (9) ≥1.450
	Lithology	7	(1) Lower Triassic; (2) Middle Triassic; (3) Upper Triassic; (4) Triassic; (5) Middle-Lower Jurassic; (6) Middle Jurassic; (7) Upper Jurassic
Meteorological and hydrological factors	Average annual rainfall (mm)	9	(1) ≤117.0; (2) 117.0~119.2; (3) 119.2~120.7; (4) 120.7~122.3; (5) 122.3~124.0; (6) 124.0~125.8; (7) 125.8~127.7; (8) 127.7~129.9; (9) ≥129.9
	Aridity	9	(1) ≤0.808; (2) 0.808~0.828; (3) 0.828~0.852; (4) 0.852~0.881; (5) 0.881~0.907; (6) 0.907~0.927; (7) 0.927~0.948; (8) 0.948~0.971; (9) ≥0.971
	Temperature (°)	9	(1) ≤16.214; (2) 16.214~16.889; (3) 16.889~17.401; (4) 17.401~17.807; (5) 17.807~18.139; (6) 18.139~18.431; (7) 18.431~18.715; (8) 18.715~19.048; (9) ≥19.048
Environmental factors	Distance from fault (m)	6	(1) ≤1000; (2) 1000~2000; (3) 2000~3000; (4) 3000~4000; (5) 4000~5000; (6) ≥ 5000
	Distance from roads (m)	6	(1) ≤10; (2) 10~20; (3) 20~30; (4) 30~40; (5) 40~50; (6) ≥ 50
	Distance from rivers (m)	6	(1) ≤100; (2) 100~200; (3) 200~300; (4) 300~400; (5) 400~500; (6) ≥500
Building factors	Building structure	7	(1) Timber structure; (2) Simple structure; (3) Adobe–timber structure; (4) Brick–timber structure; (5) Brick–concrete structure; (6) Hybrid structure; (7) Steel and reinforced concrete structure
	Construction time	7	(1) before 1939; (2) 1940~1949; (3) 1950~1959; (4) 1960~1969; (5) 1970~1979; (6) 1980~1999; (7) after 2000;
	Building storey	8	(1) 1; (2) 2; (3) 3; (4) 4; (5) 5; (6) 6; (7) 7; (8) ≥8;
	Building category	5	(1) Residential building; (2) Commercial building; (3) Teaching building; (4) Auxiliary building; (5) Other building

**Table 4:** Three-classification confusion matrix.

		Predicted Grade		
		I	II	III
**Actual grade**	I	*N_11_*	*N_12_*	*N_13_*
	II	*N_21_*	*N_22_*	*N_23_*
	III	*N_31_*	*N_32_*	*N_33_*

**Table 5:** RF and SVM optimization model evaluation indexes for the test samples.

	Accuracy	Precision			Recall			F_1_ score		
		Ⅰ	Ⅱ	Ⅲ	Ⅰ	Ⅱ	Ⅲ	Ⅰ	Ⅱ	Ⅲ
RF	97.4%	100%	95.9%	97.5%	100%	93%	98.6%	100%	94.4%	98.0%
SVM	97.4%	100%	94.9%	97.9%	100%	94%	98.2%	100%	94.5%	98.0%
Difference	0	0	1%	0.4%	0	1%	0.4%	0	0.1%	0

**Table 6:** Ranking the importance of impact factors.

Category	Impact Factors	Value of MDA	Score of MDA	Value of MDG	Score of MDG	Score of MDA and MDG	Comprehensive Ranking
Geographical and geological factors	Elevation	27.15	4	6.81	2	6	10
	TRI	86.78	11	91.35	11	22	2
	Lithology	37.78	7	11.50	3	10	8
Meteorological and hydrological factors	Average annual rainfall	18.16	3	5.24	1	4	12
	Aridity	44.21	9	16.84	7	16	4
	Temperature	42.91	8	13.10	6	14	5
Environmental factors	Distance from roads	11.72	1	12.02	4	5	11
	Distance from rivers	33.80	6	13.00	5	11	7
Building factors	Building structure	91.26	12	170.82	12	24	1
	Construction time	33.02	5	50.37	9	14	5
	Building storey	16.66	2	23.27	8	10	8
	Building category	56.82	10	68.79	10	20	3

## Data Availability

Data sharing not applicable.
